# INDIVIDUALISTIC VALUES AND SUPPORT PROVIDED TO GRANDCHILDREN

**DOI:** 10.1093/geroni/igae098.1484

**Published:** 2024-12-31

**Authors:** Tianqi Zhou, Martin Lakomý, Merril Silverstein

**Affiliations:** Syracuse University, Syracuse, New York, United States; Mendel University, Brno, Jihomoravsky kraj, Czech Republic; Syracuse University, Syracuse, New York, United States

## Abstract

The significance of individual values in family dynamics is widely acknowledged, but the role of individualism in shaping grandparental support remains insufficiently understood. In this study, we investigate the relationship between individualistic/collectivistic value orientation and two types of grandparental support (instrumental and emotional) provided by older individuals. We use data from waves 4-10 of the multigenerational Longitudinal Study of Generations to examine this link between values and support. Multilevel random-intercept models use all available respondents with the youngest grandchild aged 0-16, which makes a sample of 1309 older people nested in 307 families. Controlling for grandparents’ sex, ethnicity, marital status, health, and retirement, we found that individualistic value orientation influences instrumental support, while its impact on emotional support was not evident. Additionally, instrumental grandparental support tends to manifest more prominently within families featuring younger grandparents and younger grandchildren. In contrast, emotional support is more prevalent in families with highly educated grandparents, as well as those with more and older grandchildren. Our study complements previous studies on the relationship between values and intergenerational support by showing that individualism exerts an influence on specified forms of support such as transportation or shopping, help when sick, and childcare. These findings demonstrate the enduring presence of caregiving provided to grandchildren motivated by grandparental norms or religiosity within American families and – at the same time – reveal that the value changes can reshape the content and forms of grandparental care.

